# Characteristics of Biomechanical and Physical Function According to Symptomatic and Asymptomatic Acetabular Impingement Syndrome in Young Adults

**DOI:** 10.3390/healthcare10081484

**Published:** 2022-08-07

**Authors:** Junyong Zhang, Yonghwan Kim, Moonyoung Choi, Cong Zhang

**Affiliations:** 1 Sports Ministry, Henan University of Economics and Law, Zhengzhou 450046, China; 2 Department of Physical Education, Gangneung-Wonju National University, Gangneung 25457, Korea; 3 Department of Sports Science Convergence, Dongguk University, Seoul 04620, Korea; 4 Department of Physical Education, Yongin University, Yongin 17092, Korea

**Keywords:** dynamic balance, femoroacetabular impingement, range of motion, strength

## Abstract

Femoroacetabular impingement (FAI) is caused by hip joint anomalies. Although asymptomatic and symptomatic FAI have been reported in young adults, information on biomechanical and functional characteristics of FAI is rare. We compared the subjective hip score, range of motion (ROM), dynamic balance, and hip strength between symptomatic FAI (FAIsym) and asymptomatic FAI (FAIasym) groups and healthy controls. Participants (*n* = 307; men: 155, women: 152) were classified according to morphological abnormalities and hip joint symptoms, comprising symptomatic FAI, asymptomatic FAI, and healthy controls. The Copenhagen Hip and Groin Outcome Score (HAGOS), hip ROM, Y-balance test (YBT), and isokinetic hip strength were measured. The types of FAI were not significantly differenent in both men and women. FAIsym exhibited significantly reduced HAGOS, whereas FAIasym showed no significant difference compared to the healthy group (men: healthy 91.7 vs. FAIasym 87.2 vs. FAIsym 49.9, women: healthy 91.7 vs. FAIasym 86.2 vs. FAIsym 53.9). Hip flexion, adduction, and internal and external rotation ROMs were only significantly reduced in symptomatic FAI. Asymptomatic and symptomatic FAI groups displayed significantly lower YBT scores than healthy controls (men healthy: 84.9 vs. FAIasym: 69.0 vs. FAIsym 58.7, women healthy 79.2 vs. FAIasym 64.0 vs. FAIsym 55.5). Isokinetic hip flexion, adduction, and abduction strengths were significantly lower in FAIsym. In conclusion, FAIasym showed no decrease in muscle strength but displayed reduced dynamic balance. Subjective satisfaction, ROM, muscle strength, and dynamic balance were lower in FAIsym compared to FAIasym and healthy groups.

## 1. Introduction

**Figure 1:**
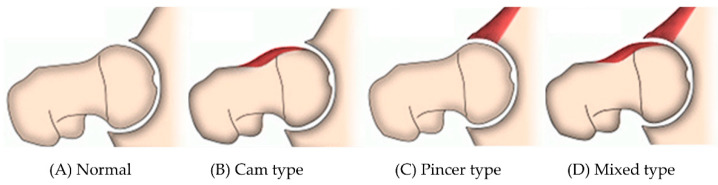
Normal and femoroacetabular impingement type.

The hip joint is a representative ball-and-socket joint in which the femoral head is inserted into the socket of the acetabulum; the shape of the bone and the capsular ligament stabilizes the structure. Owing to the structural and functional characteristics of the joint, the range of motion (ROM) is large, and various movements are possible [1]. Femoroacetabular impingement (FAI) in the hip joint occurs at the junction of the femur and acetabulum due to morphological abnormalities in the proximal femur or acetabular rim [2]. These morphological abnormalities present as deformations of the femoral head (cam type) or acetabular rim (pincer type), and FAI occurs either as individual types or as a mixture of both (Figure 1) [3,4,5].

In a large cohort study, Hale et al. [6] confirmed that 38% of patients admitted to the hospital for hip pain were diagnosed with FAI, with a reported overall annual incidence of 54.4 cases per 100,000 person years, and cam-type FAI is more common [7]. The cause of the morphological abnormalities of the hip joint that results in FAI has not been identified, and the prevalence is generally higher in men than in women [8]. It appears to be detected mainly between the ages of 20 and 30, and the timing and underlying mechanism remain largely unknown [9].

The FAI should be taken seriously because it has the potential to lead to other diseases. FAI causes chondrolabral lesions due to repetitive pathological contact between the abnormal femoral head and acetabulum, which could lead to early labral and chondral damage [10]. Melugin et al. [9] reported that in 952 patients diagnosed with FAI who were followed up for an average of 24.7 years, symptomatic hip osteoarthritis occurred in 14% of all patients and total hip arthroplasty was performed in 4%.

Typical treatment seeks to improve the pathological contact by molding the femoral head and acetabulum in the area where the contact occurs through arthroscopic surgery [11]. However, patients with mild symptoms who do not require surgery may be managed using conservative treatment, such as rest, anti-inflammatory drugs, and rehabilitation [12]. A prospective study reported that only conservative treatment significantly improved pain and function in patients with FAI [13]. Rehabilitation training consisting of muscle strength and stretching cannot induce bone morphological changes, but it can increase pain tolerance through muscle strength enhancement and improve the flexibility of soft tissues related to the hip joint ROM and subjective symptoms using a questionnaire [14,15].

Most studies analyzing the kinematic characteristics of FAI are related to ROM, but some studies have measured muscle strength and dynamic balance [16,17]. Compared with the control group, FAI patients external rotation of ROM decreased by 23–28%, and muscle strength decreased by 34% in extension, 25% in flexion, and 33% in adduction compared to the control group [16]. In addition, in the study that measured dynamic balance, there was no significant difference in the anterior in the FAI group compared to the control group, but posteromedial and posterolateral were significantly lower in the FAI group [17]

Despite this, studies on biomechanical and physical function with FAI are very rare, the number of patients participating in previous studies is small, men and women are not distinguished, and studies on asymptomatic FAI are still not covered. Additionally, there is a limitation that ROM, muscle strength, and dynamic balance are not comprehensively addressed. Moreover, considering that the morphological abnormalities of the hip joint and the age at which FAI is identified are mainly during the active ages of 20–30 years, it is very important to evaluate the functional characteristics.

Therefore, this study aimed to analyze the biomechanical and functional characteristics of patients with symptomatic and asymptomatic FAI by comparing the ROM, isokinetic hip muscle strength, dynamic balance, and subjective hip scores with those of healthy controls. We sought to enhance the understanding of field therapists regarding the physical and functional characteristics of FAI in patients, thereby contributing to the establishment of safe and effective rehabilitation training programs and treatment strategies.

## 2. Materials and Methods

### 2.1. Experimental Design

Recruitment of participants was announced on bulletin boards of hospitals, websites, rehabilitation centers, and health care centers. The participants voluntarily contacted the researcher, and the participants ranged from adults in their 20s to 30s who regularly participated in recreational sports activities such as soccer, swimming, cycling, and tennis. Researchers provided participants with information about the study process and obtained written informed consent prior to enrollment. Through consultation, the researcher recorded the participants’ history of hip or lower extremity injuries and pain and explained the benefits and potential risks associated with participating in the study, as well as the details of the examination to be performed. Subjective scores using X-ray, questionnaires, ROM, dynamic balance, and muscle strength using isokinetic equipment were measured for consenting participants.

After the examinations were completed, the orthopedic surgeon classified and analyzed the healthy group and asymptomatic and symptomatic FAI groups based on radiological findings and symptoms. This study complied with the tenets of the Declaration of Helsinki and was approved by the institutional review board of Gangneung-Wonju University (approval number: GWNU IRB 2021-13).

### 2.2. Participants

The sample size was calculated using the G*power program (G*power 3.1, University of Düsseldorf, Düsseldorf, Germany). The conditions were as follows; F test; ANOVA and one way; Effect size f = 0.25, α err prob = 0.05, power (1-β err prob) = 0.80 [18].

**Figure 2:**
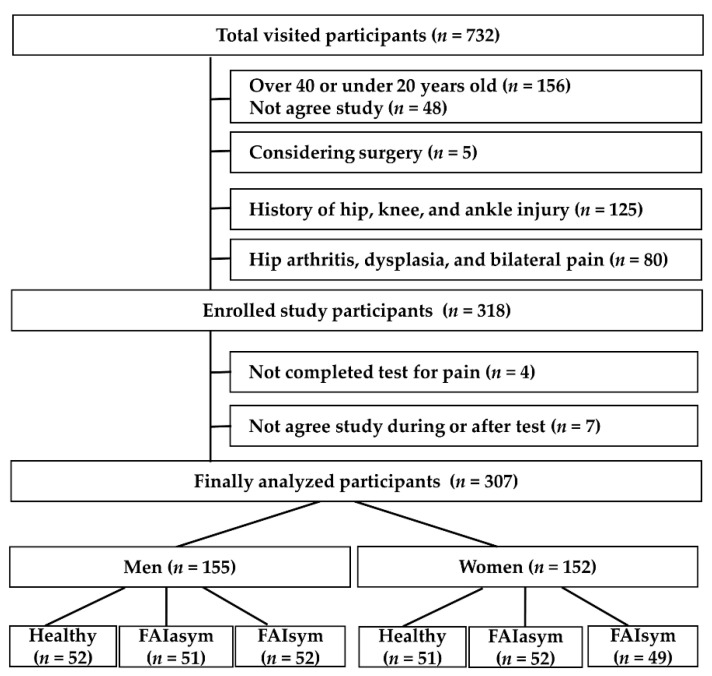
Participant’s exclusion and inclusion process.

According to the above conditions, we continued to recruit until a suitable number of participants was reached. Although the total number of visitors was 732, there were 414 individuals who met the exclusion criteria, and 318 were enrolled in the study. Finally, 11 patients were excluded because of poor physical condition, pain, and refusal to participate in the study due to changing their minds. A total of 307 patients (155 men and 152 women) were included in the final analysis. The exclusion criteria were as follows: patients who required surgery for severe pain; those with bilateral pain; history of treatment for the hip, knee, and ankle; patients who cannot complete the Y-balance test (YBT) and muscle strength tests; those over 40 or under 20 years of age (Figure 2).

The FAI diagnosis was evaluated by an orthopedic surgeon based on radiography, physical examination, pain scale, and ROM. The healthy group included participants who were normal in all tests and had no pain. The FAI in the asymptomatic group was notable on X-rays, but the patients experienced no pain. The symptomatic FAI group included patients with significant symptoms.

### 2.3. Subjective Hip Score

The hip score related to the patient’s subjective symptoms and function was determined using the Copenhagen Hip and Groin Outcome Score (HAGOS) [19]. The HAGOS is a standardized self-report questionnaire for subjectively quantifying perceived pain, functional ability, activity, and quality of life [20]. The HAGOS is a clinically questionnaire for subjective self-assessment for physically active patients with hip pain and has proven reliability and validity [20]. The questionnaire included pain (10 items), symptoms (7 items), physical function in daily living (5 items), physical function in sport and recreation (8 items), participation in physical activities (2 items), and quality of life (5 items), and comprised 6 individual subscales (total 37 items) related to the response to each question on a 5-point Likert scale, with a possible score range of 0–4. The scores of each subscale were normalized to a perfect score of 100, and the average score of the six subscales was calculated. A score of 100 indicates no problems, and lower scores indicate worse conditions.

### 2.4. Range of Motion

Hip joint ROM was measured using a manual goniometer. Flexion and extension were measured in different postures, in the supine and prone positions, respectively. The goniometer set the greater trochanter as a reference point so that the stationary arm was aligned with the lateral midline of the pelvis, and the movement arm was aligned with the lateral midline of the femur. Natural knee flexion was permitted during hip flexion. Adduction and abduction were measured with the patient in the supine position. The goniometer was set with the anterior superior iliac spine (ASIS) as the reference point, with the stationary arm oriented toward the contralateral ASIS and the movement arm aligned with the anterior midline of the femur. Internal and external rotations were measured with 90° knee flexion with the participant in the sitting position. The goniometer was set with the anterior aspect of the patella as a reference point so that the stationary arm was aligned perpendicular to the floor and the movement arm aligned with the anterior midline of the tibia. For each measurement, the angle of the endpoint of the patient’s maximum active ROM was recorded, and the higher value was recorded twice. If the error exceeded 3°, c was performed.

### 2.5. Y-Balance Test

To measure dynamic balance ability, YBT equipment (Y Balance Test™, Cerder Park, TX, USA) was used [21]. After the correct examination postures and sequences of movements were demonstrated by an experienced examiner, the sufficient practice was provided to the participants. After practice, the participants placed their feet on the stance plate at the center of the equipment and assumed a single-leg stance for examination. While maintaining balance, the opposite leg was extended as far as possible, and the tip of the foot pushed the reach indicator in the anterior (ANT), posteromedial (PM), and posterolateral (PL) directions. Remeasurement was performed when the outstretched leg touched the ground or when the heel of the standing leg fell off the stance plate. A series of motions in three separate directions was measured twice for each lower extremity, and the higher reading was analyzed. The measured absolute reach distance was recorded in centimeters and normalized as a percentage by applying leg length. Leg length was measured in centimeters as the distance between the ASIS of the pelvis and the medial malleolus of the ankle. The total score for the three separate directions was calculated as follows:

YBT score = (sum of the three reach directions/three times the limb length) × 100.

### 2.6. Isokinetic Hip Strength

Hip strength was measured using an isokinetic dynamometer (Humac Norm CSMi; Stoughton, MA, USA) to measure hip joint flexion, extension, abduction, and adduction. An isokinetic dynamometer is a mechanical device that controls speed to enable movement at a constant speed according to a set computer program. The participants actively resisted and exerted their strength using this equipment, and the detected strength was stored as graphs and numbers [22]. To maintain a consistent examination posture, the participants lay on the examination table and matched the axis of rotation of the dynamometer with the axis of the hip joint. The axis of rotation was aligned with the greater trochanter with the participant in the supine position when evaluating flexion and extension and aligned with the anterior superior iliac spine in the side-lying position when evaluating adduction and abduction.

To limit the involvement of other body parts during the examination, the contralateral leg, pelvis, and torso were fixed with straps, and a dynamometer hip attachment was attached to the distal thigh above the knee. Concentric contractions of the hip flexors, extensors, adductors, and abductors were performed at an angular velocity of 30 °/s. To help participants understand, an experienced examiner provided adequate explanations and allowed the participants to practice several times before proceeding. The hip joint movement angle for examination was set at 0–100° for flexion and extension tests and 0–45° for adduction and abduction tests. The participants performed the main test four times after three practice movements. Considering the effect of the weight of the lower extremity segment, the lever arm was placed as close as possible to the horizontal position, and gravity correction was performed. The measured peak torque was recorded in Nm, and for analysis, the value was divided by body weight (Nm/kg) and adjusted as a percentage.

### 2.7. Data Analysis

Data analysis was performed using SPSS 22.0 (IBM SPSS Inc., Chicago, IL, USA). Normality tests were performed using the Kolmogorov–Smirnov and Shapiro–Wilk tests. The main variable was not normally distributed; therefore, a nonparametric test was performed. The Mann–Whitney test was used for comparisons between the two groups, and the Kruskal–Wallis test and Bonferroni post hoc test were performed to compare the three groups. In addition, the chi-square test was performed on categorical variables for FAI type occurrence. Statistical significance was set at *p* < 0.05.

## 3. Results

### 3.1. General Characteristics of Participants

Participants were classified according to their group, and their general characteristics are presented in Table 1. Age, height, weight, and body mass index were not significantly different among the groups. There were no significant differences in FAI type according to the presence or absence of symptoms in men and women.

### 3.2. Subjective Hip Score

Table 2 show differences in the HAGOS scores measured to evaluate the subjective hip score between the groups. Symptoms, pain, ADL, sports and recreation, physical activity, quality of life, and total scores were significantly lower in symptomatic FAI than in asymptomatic FAI and healthy groups, in both men and women. There was no significant difference in the scores between the asymptomatic FAI and healthy groups in both sexes.

### 3.3. Range of Motion

Table 3 show the differences in hip ROM between the groups. The symptomatic FAI group showed significantly reduced hip flexion, abduction, and internal and external rotation ROMs in both men and women compared with asymptomatic FAI and healthy groups. However, there was no significant difference in extension and abduction between the sexes. There was no significant difference in ROM between men and women in the asymptomatic FAI and healthy groups.

### 3.4. Dynamic Balance

**Figure 3:**
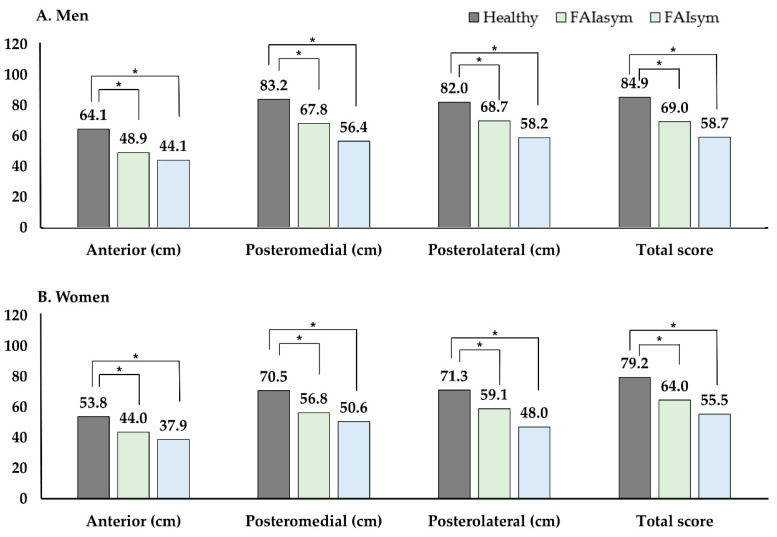
Comparison of Y-balance test in healthy, asymptomatic and symptomatic femoroacetabular impingement patients. * *p* < 0.05, significance. Abbreviations: FAI, femoroacetabular impingement; sym, symptomatic; asymptomatic, asymptomatic.

Figure 3 show the differences in YBT measured to evaluate dynamic balance among the groups. Both the symptomatic and asymptomatic FAI exhibited significantly lower scores in the anterior, posteromedial, and posterolateral reach distances and total scores than the healthy groups of both sexes.

### 3.5. Isokinetic Hip Strength

**Figure 4:**
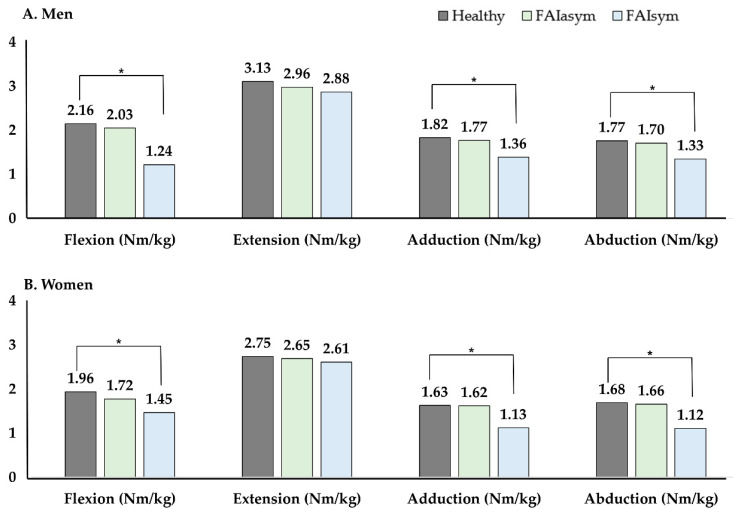
Comparison of isokinetic strength in healthy, asymptomatic and symptomatic femoroacetabular impingement patients. * *p* < 0.05, significance. Abbreviations: FAI, femoroacetabular impingement; sym, symptomatic; asym, asymptomatic; Nm, Newton meter.

The differences in isokinetic hip strength between groups are illustrated in Figure 4. The symptomatic FAI group demonstrated significantly lower muscle strength for hip flexion, adduction, and abduction than the asymptomatic FAI and healthy groups, in both men and women. However, there was no significant difference in extension between men and women. No significant difference in muscle strength was observed between the asymptomatic FAI and healthy groups of both sexes.

## 4. Discussion

FAI has been reported as one of the causes of hip pain in active young adults [23,24]. However, the literature states that FAI is not necessarily accompanied by pain [25]. Our findings revealed that the subjective hip joint score evaluated by the HAGOS was significantly lower in the symptomatic FAI group than in the healthy group, whereas the asymptomatic FAI group was not significantly different from the healthy group. This means that FAI is not necessarily related to pain, and the radiological finding showed that 29.6% of the adult population had asymptomatic FAI [26,27].

The deep socket of the acetabulum of the pelvis and large spherical femoral head provide the hip joint with the advantage of a large ROM, but the disadvantage is that the surface area of the bone is large. Therefore, abnormal contact between the femoral neck and acetabular rim impairs hip kinematics and causes pain [16]. Hip joint ROM is the most common clinical parameter for diagnosing hip joint pathological conditions, such as osteoarthritis and FAI, and for monitoring therapeutic effects [28].

Most studies focusing on hip ROM have reported that patients with FAI exhibit lower hip flexion, adduction, and rotation ROM than healthy controls [16,29,30]. However, there is insufficient evidence regarding the characteristics of hip ROM in the asymptomatic FAI group. In this study, the symptomatic FAI group showed significantly reduced hip flexion, abduction, and internal and external rotation ROMs compared to the healthy group. The ROM characteristics of the symptomatic patients observed in this study were consistent with those reported in previous studies. On the other hand, the asymptomatic FAI group did not show a significant difference in ROM compared to the healthy group, even though they were diagnosed with morphological abnormalities, similar to the symptomatic FAI group.

Differences in ROM between the symptomatic and asymptomatic FAI groups are believed to be related to symptoms and pain. A previous study also attempted to identify the causes of limited ROM in patients with FAI. Naili et al. [31] analyzed the association between hypothetical ROM measured using computed tomography motion simulation and maximum passive ROM using three-dimensional motion analysis. The authors found that hip ROM in patients with FAI was more limited by pain than morphology-based impingement.

A decrease in dynamic balance ability is associated with a decrease in the function of dynamic single-leg balance control, which may decrease the control of dynamic movement, affecting the biomechanics of gait [32]. Decreased balance is one of the main symptoms commonly observed in patients with soft tissue damage and osteoarthritis [33]. A decrease in dynamic balance ability was reported in FAI [30,32]. Freke et al. [30] confirmed through a systematic review that patients with symptomatic FAI showed reduced dynamic balance in a single-leg stance. Hatton et al. [32] reported a decrease in the dynamic hip stability related to neuromuscular control in patients with symptomatic FAI.

In this study, dynamic balance was evaluated using YBT. Interestingly, dynamic balance was significantly lower in the symptomatic FAI group than in the asymptomatic FAI group. However, no significant difference was observed between the symptomatic and asymptomatic FAI groups. The decrease in dynamic balance ability of symptomatic and asymptomatic patients may be due to the possibility of microlabral injury. The acetabular labrum is rich in nerve end organs that play an important role in proprioceptive feedback related to dynamic balance [34]. Therefore, the labrum potentially modulates hip proprioception and damage to the labrum may lead to impaired proprioceptive feedback. Although FAI may be asymptomatic, micro-labral injury due to morphological abnormalities could be a potential cause of loss of dynamic balance [35].

Several previous studies attempted to objectively evaluate the relationship between biomechanics and hip muscle strength in patients with FAI [36,37,38]. The authors reported that they found kinematic changes in the lower extremities that were symptomatic during dynamic weight-bearing activities and that these changes were related to weakness of the hip joint muscles. Ng et al. [36] investigated muscle contributions and hip contact forces in patients with symptomatic FAI and found altered gait kinematics and dynamics with a decrease in the hip flexor muscle force and anterosuperior hip contact force. Spiker et al. [37] compared lower extremity kinematics, kinetics, and hip muscle electromyography activity between patients with FAI and healthy controls and reported an increase in knee dynamic valgus and a decrease in hip abductor muscle activity during walking and stair climbing.

In a study by Casartelli et al. [39], patients with symptomatic FAI showed significantly lower muscle strength in hip flexion (26%), adduction (28%), abduction (11%), and external rotation (18%) than the healthy controls. In this study, the symptomatic FAI group showed significantly lower isokinetic hip strength for flexion, adduction, and abduction than the healthy group. Our study revealed that the results for symptomatic FAI were consistent with the findings of previous studies. A major finding of the present study was that the asymptomatic FAI group showed no significant difference in muscle strength compared to the healthy group. Previous studies reported that FAI-related muscle weakness may result from various factors such as mechanical limitations within the hip joint, decreased muscle mass, and inhibition of muscle activation [39,40]. Hip muscle weakness may lead to changes in lower-extremity kinematics during dynamic weight-bearing activities, and these kinematic changes may be potential factors for dysfunction and pain reported by symptomatic FAI patients.

If there is a lesion or injury to the hip joint, it results in functional limitations, including difficulties in ADLs such as walking, wearing pants, sitting and standing up, driving, tying shoelaces, and ascending and descending stairs. The onset of symptomatic hip disorders in non-arthritic hip joints is associated with the anatomical structure and morphology of the hip joint, repetitive mechanical loads, and acute injuries during daily activities and sports [3]. However, according to Siebenrock et al. [25], hip joint morphological abnormalities are mainly developmental deformities caused by high-intensity sports activities during growth, but they are not necessarily accompanied by pain; therefore, even if FAI occurs, it may be asymptomatic.

Dynamic and static factors affecting hip biomechanics and function are complex, but the most common morphological abnormalities are loss of femoral head-neck offset (cam type) and focal or global acetabular over coverage (pincer type) [4]. These anatomical abnormalities cause repeated impingement during dynamic hip movement, which can lead to loading of the femoral head–neck junction to the acetabular rim, labral injury, cartilage dissection, and premature degenerative changes [41].

Therefore, many cases of FAI have been reported after being asymptomatic during adolescence. A study investigating the prevalence of FAI in asymptomatic individuals (average age: 25 years) showed that the prevalence of cam and pincer deformities was 37 and 67%, respectively. In addition, the rate of cam deformity was three times higher in athletes than in non-athletes, and the prevalence of labral injury was 68%, despite being asymptomatic [35].

Early diagnosis is important, even if asymptomatic, because FAI causes pathological damage to the hip joint and is considered a precursor symptom of osteoarthritis [28]. This study was conducted to enhance the understanding of FAI by field experts, such as rehabilitation specialists. Even if the patient is asymptomatic, balance is lost. Therefore, improvement of dynamic stabilization through lower-extremity neuromuscular system training rather than muscle strength training during rehabilitation and prevention of secondary injury should be planned. A limitation of this study is that the groups were divided using only symptoms or asymptomatic factors due to FAI, and subdivision based on the severity of the disease was not conducted. There were no age ranges, and the number of participants was relatively small. In addition, the differences in strength and balance ability according to the amount of daily exercise or physical activity were not considered. Although FAI reported that symptoms improved after 14 weeks of strength and stretching training [15], FAI rehabilitation research is still limited. In the future, our research will need to develop an effective FAI rehabilitation program through more precise training interventions.

## 5. Conclusions

In this study, the symptomatic FAI group exhibited decreased subjective hip score, hip ROM (flexion, abduction, and internal and external rotations), dynamic balance, and hip muscle strength (flexion, adduction, and abduction) compared to the healthy group. In contrast, the asymptomatic FAI group showed reduced function compared to the healthy group only in dynamic balance. Therefore, even in asymptomatic FAI patients, management is required to prevent the potential risk of pathological damage to the hip joint and the development of osteoarthritis. Efforts to improve dynamic balance are recommended.

## Figures and Tables

**Table 1:** General characteristics of participants.

Variables	Men				Women			
	Healthy (*n* = 52)	FAIasym (*n* = 51)	FAIsym (*n* = 52)	*p*-Value	Healthy (*n* = 51)	FAIasym (*n* = 52)	FAIsym (*n* = 49)	*p*-Value
Age, years	28.4 ± 3.3	27.2 ± 4.5	28.5 ± 3.6	0.241	27.1 ± 4.5	26.2 ± 3.3	26.7 ± 3.4	0.457
Height, cm	176.5 ± 4.5	177.4 ± 4.6	176.3 ± 3.3	0.563	166.3 ± 3.2	167.0 ± 3.2	165.4 ± 4.2	0.410
Weight, kg	70.3 ± 5.4	69.7 ± 5.3	71.7 ± 4.5	0.674	58.7 ± 6.6	59.3 ± 7.4	57.3 ± 6.5	0.419
BMI, kg/m^2^	22.7 ± 3.7	22.3 ± 3.7	23.1 ± 3.8	0.301	21.4 ± 2.3	21.5 ± 3.5	21.2 ± 3.3	0.386
Cam type	–	21 (41.2%)	22 (42.3%)	0.360	–	23 (44.2%)	20 (40.8%)	0.312
Pincer type	–	17 (33.3%)	14 (26.9%)		–	16 (30.8%)	15 (30.6%)	
Mixed type	–	13 (25.5%)	16 (30.8%)		–	13 (25.0%)	14 (28.6%)	

*p* < 0.05; Abbreviations: FAI, femoroacetabular impingement; asym, asymptomatic; sym, symptomatic; BMI, body mass index.

**Table 2:** Participant’s hip score using questionnaire.

HAGOS	Men				Women			
	Healthy	FAIasym	FAIsym	*p*-Value	Healthy	FAIasym	FAIsym	*p*-Value
Symptoms	91.3 ± 3.8	85.2 ± 7.5	55.4 ± 18.5 ^b,c^	<0.001	93.5 ± 2.2	86.6 ± 8.7	64.5 ± 17.5 ^b,c^	<0.001
Pain	93.2 ± 4.6	90.5 ± 8.4	61.3 ± 23.3 ^b,c^	<0.001	90.6 ± 2.4	88.4 ± 9.5	53.4 ± 16.7 ^b,c^	<0.001
ADL	92.4 ± 4.3	90.7 ± 6.2	56.6 ± 19.8 ^b,c^	<0.001	90.8 ± 2.2	88.7 ± 7.8	58.7 ± 21.4 ^b,c^	<0.001
Sport and recreation	89.6 ± 5.7	85.8 ± 8.8	38.1 ± 21.9 ^b,c^	<0.001	88.3 ± 4.6	85.1 ± 7.9	44.2 ± 13.7 ^b,c^	<0.001
Physical activity	93.2 ± 2.1	87.9 ± 8.0	40.8 ± 26.0 ^b,c^	<0.001	89.9 ± 3.8	87.8 ± 8.0	38.9 ± 18.2 ^b,c^	<0.001
Quality of life	91.6 ± 3.2	86.6 ± 5.6	43.4 ± 26.4 ^b,c^	<0.001	91.2 ± 3.9	85.4 ± 8.4	61.5 ± 14.8 ^b,c^	<0.001
HAGOS total score	91.7 ± 4.2	87.2 ± 7.4	49.9 ± 22.2 ^b,c^	<0.001	91.7 ± 2.4	86.2 ± 8.2	53.9 ± 17.3 ^b,c^	<0.001

*p* < 0.05; significance, ^b^ = FAIasym versus FAIsym; ^c^ = Healthy versus FAIsym; Abbreviations: HAGOS, hip and groin outcome scale; FAI, Femoroacetabular impingement; sym, symptomatic; asym, asymptomatic; ADL, activities of daily living.

**Table 3:** Participant’s hip range of motion.

ROM, Degree	Men				Women			
	Healthy	FAIasym	FAIsym	*p*-Value	Healthy	FAIasym	FAIsym	*p*-Value
Flexion	130.0 ± 12.6	126.6 ± 14.3	108.6 ± 20.7 ^b,c^	0.021	138.3 ± 14.7	132.5 ± 15.5	109.3 ± 18.3	0.004
Extension	13.4 ± 3.4	12.3 ± 3.2	13.1 ± 3.2	0.385	15.2 ± 5.4	14.8 ± 5.3	15.2 ± 5.5	0.320
Adduction	22.6 ± 3.8	21.9 ± 3.8	19.7 ± 6.2 ^b,c^	0.016	24.7 ± 6.2	22.5 ± 6.8	20.6 ± 8.6 ^b,c^	0.003
Abduction	39.8 ± 5.2	38.5 ± 6.0	38.3 ± 6.7	0.127	44.4 ± 9.1	42.3 ± 9.5	41.7 ± 8.7	0.358
Internal rotation	43.4 ± 5.9	40.2 ± 7.4	35.6 ± 5.1 ^b,c^	0.004	48.3 ± 7.8	45.8 ± 8.3	38.8 ± 8.8 ^b,c^	0.012
External rotation	49.3 ± 5.3	42.9 ± 5.2	28.2 ± 6.6 ^b,c^	<0.001	53.8 ± 8.3	47.3 ± 10.7	33.5 ± 9.5 ^b,c^	<0.001

*p* < 0.05; ^b^ = FAIasym versus FAIsym; ^c^ = healthy versus FAIsym. Abbreviations: ROM, range of motion; FAI, femoroacetabular impingement; sym, symptomatic; asymptomatic, asymptomatic.

## Data Availability

The data are not publicly available because of privacy or ethics.
